# Postprandial blood glucose response: does the glycaemic index (GI) value matter even in the low GI range?

**DOI:** 10.1038/s41387-020-0118-5

**Published:** 2020-05-01

**Authors:** Bhupinder Kaur, Melvin Koh, Shalini Ponnalagu, Christiani Jeyakumar Henry

**Affiliations:** 1 Clinical Nutrition Research Centre (CNRC), Singapore Institute of Food and Biotechnology Innovation (SIFBI), Agency for Science, Technology and Research (A*STAR), Singapore, Singapore; 2 Department of Biochemistry, Yong Loo Lin School of Medicine, National University of Singapore, Singapore, Singapore

**Keywords:** Nutrition, Type 2 diabetes

## Abstract

A growing body of research over the last decades has shown that diets based on the low glycaemic index (GI) foods reduce the risk of developing diabetes and improve blood glucose control in people with diabetes. The range of inflexion on the glycaemic response of low GI (LGI) foods is an interesting observation that has not been studied by many. LGI 1 (GI 54 ± 3.3) biscuit was formulated using a basic formulation while the LGI 2 (23.8 ± 3.3) biscuits was a modification of LGI 1 recipe, formulated with the inclusion of functional ingredients. Biscuits were formulated to be iso-caloric (kcal/100 g: 521 ± 12). Each participant consumed identical standard meals for lunch and dinner. Biscuits were consumed as breakfast and mid-afternoon snack. Using a randomized, controlled, crossover study, 13 males [(means ± SD) age: 25.3 ± 1.0 years, BMI 21.6 ± 0.5 kg/m^2^, fasting blood glucose 4.7 ± 0.1 mmol/L] wore continuous glucose monitoring systems (CGMS™) for 3 days for each test session. The postprandial glycaemic response and insulin response were compared within participants. Total iAUC for breakfast and standard dinner were significantly lower for LGI 2 treatment (*p* < 0.05) than LGI 1 treatment. Second-meal glucose tolerance was observed at the dinner meal. The overall iAUC insulin response over 180 min was significantly lower for LGI 2 biscuits (*p* = 0.01). The postprandial glycaemic response of two types of biscuits that fall within the low GI classification (GI 24 and 54) differed with LGI 2 biscuits (GI 24) showing a more suppressed postprandial glycaemic response. Our study shows that even within the low GI range, the GI value matters in influencing postprandial glucose.

## Introduction

The prevalence of type 2 diabetes (T2D) is on the rise globally^1^. The World Health Organisation estimated that 2.2 million deaths in 2012 were attributed to high blood glucose and related comorbidities, with another 1.5 million directly attributed to diabetes^2^. A holistic approach to manage the disease is recommended, including dietary modifications, increasing physical activity and pharmaceutical interventions to manage blood glucose levels if necessary. Among these, dietary modifications play a significant role in diabetes management. Blood glucose concentration is affected by factors, such as type and amount of dietary carbohydrate, nature of starch, quantity of protein and fat, dietary fibre content, particle size, method of food processing, and food form ref. ^3^. The main aim of these dietary interventions are to reduce the glycaemic index (GI) of the food so that the blood glucose does not increase after its consumption. The GI was coined by Jenkins et al.^3^. It indicates the blood glucose-raising potential of foods. Foods have been classified as being low, medium or high GI based on this concept. There is substantial evidence to suggest that consumption of low GI foods can result in a lower glycaemic response which can reduce the risk of type 2 diabetes and cardiovascular disease^4,5^. Therefore, there is an increased consumer demand for diabetes-related functional foods, with the primary goal of improving blood glucose response.

To date, there have been numerous studies that investigated the relationship between the GI of foods and the subsequent postprandial glycaemic response^5–8^. Postprandial blood glucose levels have been shown to be better predictors of long-term health consequences^9^. Thus, lowering fluctuation and peaks of blood glucose after carbohydrate meals is important. However, the majority of studies investigating the impact of GI on the postprandial glycaemic response generally compare between the low (GI > 55) and high GI (GI < 70) categories. The impact on postprandial glycaemic response between foods classified within the same range i.e. low GI (GI > 55) but with differing GI values (24 and 54) has not been reported.

Second meal effect is another factor that is studied along with the GI of the foods. Second meal effect refers to the effect of the first meal on the postprandial glycaemic response of the second meal, termed the “second meal phenomenon”^10^. Various studies have investigated this phenomenon using various GI food types^11,12^. It has been observed widely that a low GI food will reduce the subsequent postprandial glycaemic response largely as compared to a high GI food. However, such investigations have not been done among foods that belong to the same GI range i.e second meal effects of two low GI foods, which will be an interesting observation to make.

This study, for the first time, compared the postprandial glycaemic response of two types of biscuits that fall within the low GI range. Though both biscuits are classified to be low GI, the range of inflexion on the glycaemic response is an interesting observation that has not been studied by many. The aim of this study was to compare the glycaemic impact of a basic low GI biscuit (GI 54) against a modified version of this biscuit that had a lower GI (GI 24). The biscuits were tested in young, healthy non-diabetic volunteers. This study also explored the potential second meal effect after the consumption of the biscuits.

## Subjects and methods

The study was conducted in accordance with the guidelines laid down in the Declaration of Helsinki, and all procedures involving human participants were approved by the Domain Specific Review Board (DSRB) of National Healthcare Group, Singapore (Reference no. 2018/01066).

### Subjects

The inclusion criteria for participants were healthy, young Asian Chinese males aged between 21 and 40 years, non-smoker, body mass index (BMI) between 18.5 and 25 kg/m^2^ and normal blood pressure (<140/90 mm·Hg). Exclusion criteria were metabolic diseases (such as diabetes, hypertension, etc.), known glucose-6-phosphate dehydrogenase deficiency (G6PD deficiency), medical conditions and/or taking medications known to affect glycaemia (glucocorticoids, thyroid hormones and thiazide diuretics), intolerances or allergies to foods, partake in sports at the competitive and/or endurance levels, intentionally restrict food intake, and fasting blood glucose more than 6 mmol/L. A total of 14 participants were screened and recruited. One subject dropped out after one session, resulting in 13 data sets being analysed. The study was conducted at the Clinical Nutrition Research Centre (CNRC), Singapore. The protocol was well explained to the subjects and they gave their informed consent before participation. The study was registered in the Clinicaltrial.gov registry as NCT04115579.

### Study protocol

**Fig. 1:**
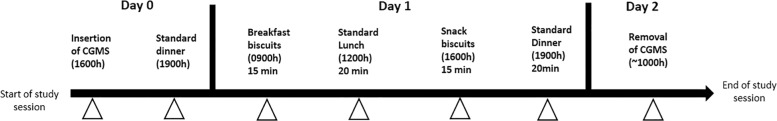
The 3-day study protocol, consisting of two sessions as a randomized, cross-over trial: all participants consume identical standard meals and biscuits, while wearing the continuous glucose monitoring (CGM) device. On day 0, CGM was inserted and a standard meal was given. On Day 1, breakfast at 09:00 h, lunch at 12:00 h, snack at 16:00 h and dinner at 19:00 h. On day 2, the CGM device was removed from participant.

A randomized, controlled, single-blinded cross-over design was adopted for this study. Each participant attended two test sessions (consisting of 3 days each), separated by a wash-out period of at least 3 days. Figure 1 shows a schematic overview of a study session. Participants were advised not to perform any rigorous activities three days prior to and during the study session. At each session, subject would consume either the LGI 1 biscuit or the LGI 2 biscuits, depending on the randomization for that session.

Each test session spanned three consecutive days from around 16:00 on day 0 till 9:00 on day 2 consisting of over 24 h continuous glucose monitoring (CGM). On day 0, the continuous glucose monitoring system (CGMS™) was inserted in the afternoon. On day 1, participants arrived at the centre around 8:30 am to 9:00 am following a 10–12 h overnight fast. The participants were first allowed to rest for 10 min before testing began. An indwelling intravenous cannula was inserted into a forearm vein by a phlebotomy-trained state registered nurse and a baseline blood sample (0 min) was obtained. Subsequently, participants consumed the LGI 1 or LGI 2 biscuits, with 250 ml water, at a comfortable pace within 15 min. Following the breakfast meal, venous blood samples were collected at 30, 60, 90, 120, 150 and 180 min intervals following the start of the meal. Participants were then given a standardized lunch consisting of spaghetti with chicken sauce and a fruit cocktail which was to be consumed in 20 min. LGI 1 or LGI 2 biscuits were given for afternoon snack to be consumed at home at 16:00 h (within 15 min) and a standardized dinner to consume at home at 19:00 h (within 20 min).

### Treatment meals

All standardized meals for lunch and dinner had the same macronutrient content and composition. These standard meals reflected a typical local rice-based or pasta-based meal accompanied with a drink or fruit. All meals given were identical for both sessions, with the only difference being the treatment biscuits given for breakfast and snack. Participants were not allowed to eat or drink anything other than the test meals and plain water during the study period. All participants were also asked to avoid alcohol and excessive physical activity for 2 days prior to and during the study period.

LGI 1 biscuits and LGI 2 biscuits were produced in the CNRC food product development kitchen. The GI of biscuits were previously tested according to the ISO 26642:2010 method, in the CNRC laboratory^13^. LGI 1 biscuits were formulated using basic ingredients for a biscuit recipe consisting of all-purpose flour, butter, sugar, vanilla flavour, baking soda, egg and salt. In the formulation of LGI 2 biscuits, all-purpose flour was replaced with a mixture of plain flour, soluble fibre and a plant-based protein (derived from soya). Butter was replaced with coconut oil and sugar was partially replaced with a low GI sweetener. LGI 1 and LGI 2 biscuits were given in portions containing 50 g of available carbohydrates at breakfast and 25 g available carbohydrates for mid-afternoon snack. The LGI 1 biscuit had a GI of 54.4 ± 6.3 and LGI 2 biscuit had a GI of 23.8 ± 3.3. Table 1 shows the nutrient composition of both biscuits, and the study foods provided for both sessions.

**Table 1:** Composition and macronutrient content of study meals.

**Day 1**	Energy (kcal)	CHO (g)	Fat (g)	Protein (g)	Fibre (g)	Available CHO (g)
*LGI 1 Treatment*						
*Breakfast*						
LGI 1 biscuits	520.7	50.5	33.2	5.0	0.5	50.0
*Lunch*						
Spaghetti with chicken sauce	535.0	97.0	8.3	17.3	3.3	93.7
Fruit cocktail						
*Snack*						
LGI 1 biscuits	260.3	25.3	16.6	2.5	0.3	25.0
*Dinner*						
Teriyaki chicken with rice	634.0	104.0	13.4	22.4	1.0	103.0
Milo drink						
*Total*	1950.3	276.8	71.4	47.2	5.1	271.7
*LGI 2 Treatment*						
*Breakfast*						
LGI 2 biscuits	520.7	57.3	27.7	10.6	7.3	50.0
*Lunch*						
Spaghetti with chicken sauce	535.0	97.0	8.3	17.3	3.3	93.7
Fruit cocktail						
*Snack*						
LGI 2 biscuits	260.4	28.7	13.8	5.3	3.7	25.0
*Dinner*						
Teriyaki chicken with rice						
Milo drink	634.0	104.0	13.4	22.4	1.0	103.0
*Total*	1950.3	287.0	63.2	55.7	15.3	271.7

*CHO* carbohydrate.

### CGM and insulin measurement

Continuous glucose monitoring (CGM) (iPro™2 Professional CGM-Medtronic MiniMed, Northbridge, CA, USA) was used to measure glycaemic response, defined as the primary outcome. The insertion was performed on day 0 around 16:00 and the sensor was removed on day 2 of the study at 9:00. During each test session, the CGM sensor was calibrated against finger-stick blood glucose measurements four times a day before every meal and before sleeping using the FreeStyle Optium Neo Blood Glucose meter (Abbott Laboratories). Data were collated and processed using online software (Medtronic Diabetes CareLink iPro; carelink.minimed.eu). The data reported in this paper represent 24 h interstitial glucose readings recorded every 5 min from the start 00:00 Day 0 until 24 h later around 00:00 on day 2.

On day 1, participants arrived in a fasted state and a finger-prick blood glucose measurement for CGM calibration was taken and this fasting blood glucose measurement was recorded. Then an indwelling intravenous cannula was inserted into a forearm vein by a phlebotomy-trained state registered nurse and a baseline blood sample (0 min) was obtained. Subsequently, participants consumed the LGI 1 or LGI 2 biscuits, at a comfortable pace and finished it within 12 min. Venous blood samples were collected at 30, 60, 90, 120, 150 and 180 min intervals following the start of the meal. Insulin determinations were performed for both LGI 1 and LGI 2 arms. Venous blood samples collected were centrifuged at 1500 × *g* for 10 min at 4 °C, and serum was aliquoted and stored at −80 °C. Serum insulin concentrations were determined using a Cobas e411 (Roche, Hitachi, USA), where the intra- and inter-assay CVs were <5% and <6%, respectively.

### Data processing and statistical analysis

The primary outcome was to determine how the inclusion of LGI 1 and LGI 2 biscuits would affect postprandial glycaemic response over the 24 h. The baseline glucose value for each subject was determined from the average CGM interstitial glucose readings for half-hour at a fasted state on day 1. It was used to calculate the change in glucose levels for the subsequent time points for the 24 h. The glycaemic response was expressed as the incremental area under the curve (iAUC) and calculated using the trapezoidal rule^14,15^. The secondary outcome was the insulin response during breakfast. The IAUC insulin was also calculated using the trapezoidal rule during the breakfast period^14,15^. All areas below baseline were excluded from the calculations.

A cross-over design with a minimum of 8 subjects would be sufficient to detect a 15% change in area under the glucose curve (24 h) with a power of 0.85 at a significance level of 0.05^6^. Data and figures were processed in a Microsoft Excel spreadsheet (Microsoft Corporation). Values were presented as mean ± SEM (standard error of the mean) unless otherwise stated, coefficient of variation (CV) was reported as median (Inter-Quartile range). Prior to statistical analysis, the normality of the data were assured using the Shapiro–Wilks test and Quantile–Quantile (Q–Q plot) of the differenced values. The parametric paired *t* test was used to compare the mean iAUC values between the treatments and non-parametric *t* test was used for the comparison of CV between the treatments. Statistical significance was set at *p*-value < 0.05. All statistical analyses were performed using Statistical Package for the Social Sciences version 23 (SPSS Inc.).

## Results

### Baseline characteristics

For the present study, 14 participants enrolled, but one was excluded because he was unable to complete the second session due to personal reasons. Thus, 13 young, healthy Chinese male adults completed both arms of the study, and their characteristics are shown in Table 2.

**Table 2:** Characteristics of study participants (*n* = 13).

Anthropometric and physiological parameters	Mean ± SD
Age (years)	25.3 ± 1.0
Height (cm)	171.7 ± 1.8
Weight (kg)	63.9 ± 2.0
BMI (kg/m^2^)	21.6 ± 0.5
Systolic blood pressure (mm Hg)	121.3 ± 2.5
Diastolic blood pressure (mm Hg)	71.7 ± 3.1
Waist circumference (cm)	74.5 ± 1.4
Hip circumference (cm)	93.6 ± 1.3
Fasting blood glucose (mmol/L)	4.7 ± 0.1

Data are means ± SD (standard deviation).

### Assessment by continuous glucose monitoring

**Fig. 2:**
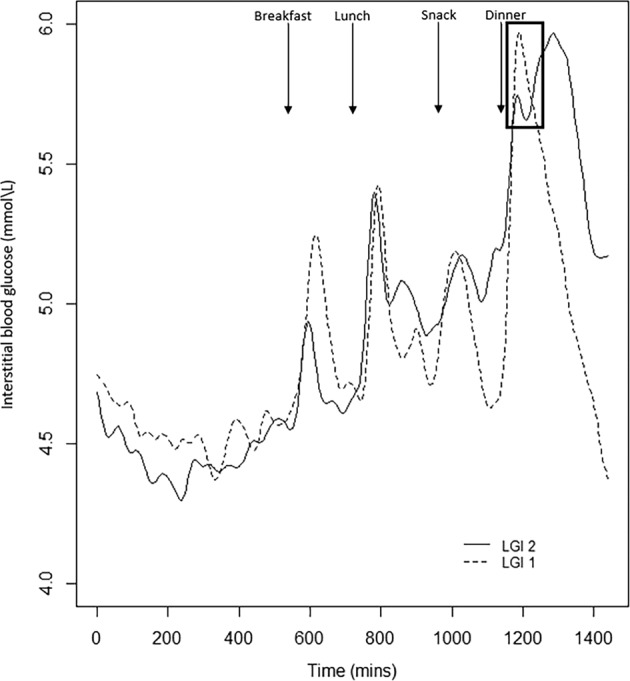
Mean 24-h continuous glucose monitoring (CGM) profiles derived from all volunteers (*n* = 13) after LGI 1 and LGI 2 treatment. The black arrows represent the average meal times. The rectangle indicates the second-meal effect.

**Fig. 3:**
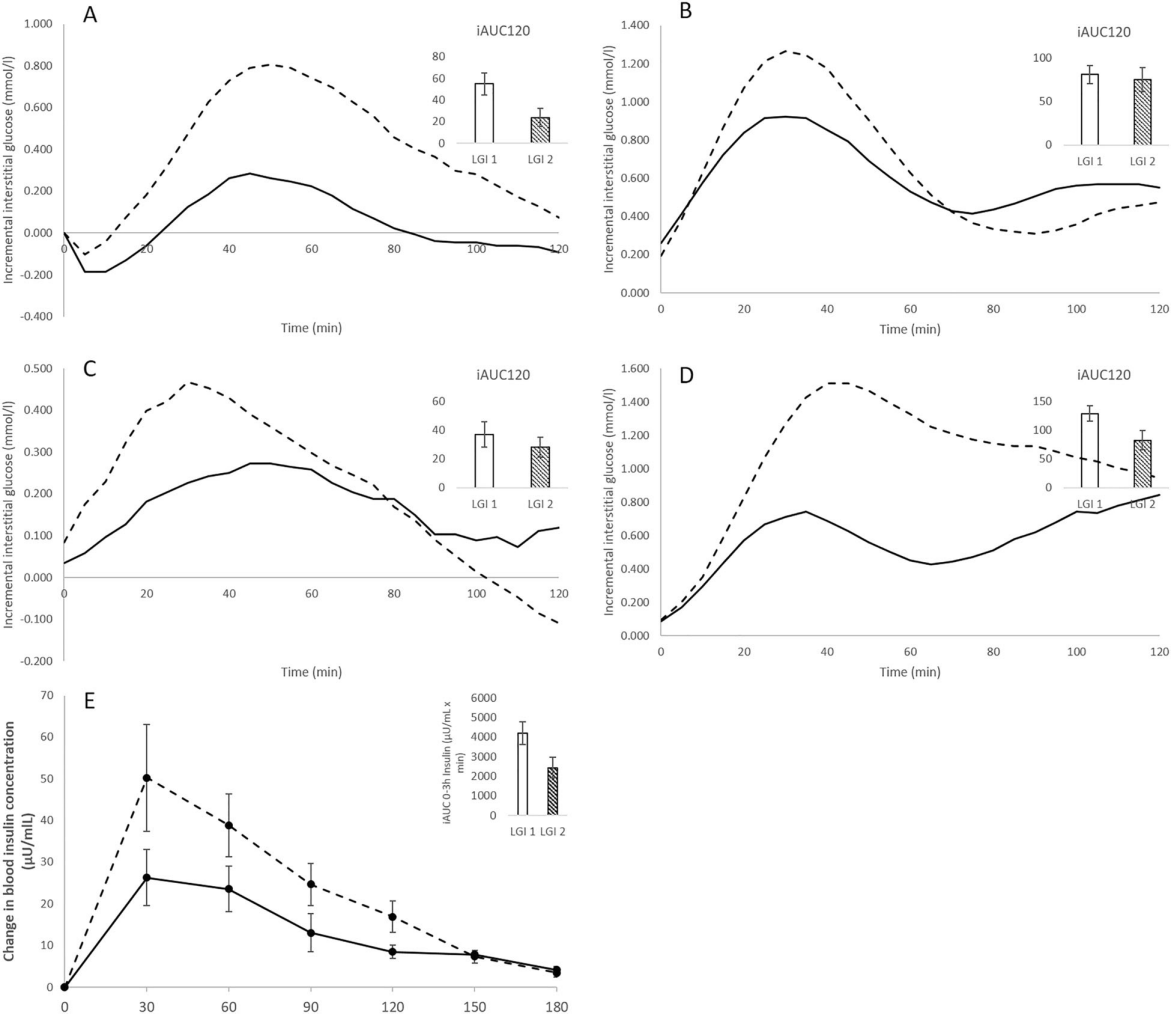
Mean postprandial glucose and insulin concentrations (incremental and iAUC) in participants (*n* = 13). **a** Represents the breakfast portion of the incremental glucose curves for 120min; **b** represents the lunch portion; **c** represents the snack portion; **d** represents dinner portion. The solid black line represents the LGI 2 biscuits and the dashed lines represent the LGI 1 biscuits. The bar plots on the right hand side are displayed as mean with error bars using SEM; *n*=13. iAUC120 was calculated using the trapezoid rule ignoring the area below the baseline. Total iAUC120 corresponds to the area under the curve for the entire 120min of measurement. **e** Represents the mean change from baseline postprandial insulin after breakfast over 180min. The iAUC for blood insulin concentration in the overall 180min after the breakfast (bar plot). *p-value<0.05 (LGI 2 biscuits compared to LGI 1 biscuits). *P* value calculated using paired *t* test.

There were no significant differences in the fasting concentrations of glucose prior to the consumption of the LGI 1 and LGI 2 biscuits at breakfast (*p*-value = 0.61). The CGM glycaemic profiles for the LGI 1 and LGI 2 treatments are graphically presented in Fig. 2. The glycaemic outcome parameters are presented in Table 3. The incremental glucose peak, iAUC 0–1 h, 0–2 h and 0–3 h were significantly lower after LGI 2 biscuits compared to the LGI 1 biscuits (*p*-value < 0.05). The LGI 2 snack had a lowered postprandial glucose response at the first 1 h that was also observed with the standard dinner (Table 3). The total iAUC120 for LGI 2 breakfast, and standard dinner were significantly lower for the LGI 2 treatment (*p*-value < 0.05) than LGI 1 treatment (Fig. 3). There was no significant difference in the median iAUC 24 h (*p*-value = 0.51), between the treatments from 12 midnight of day 0 till 12 midnight of day 2.

**Table 3:** Glucose parameters in healthy subjects (*n* = 13).

Glycaemic parameters	LGI 2	LGI 1	*P* value	LGI 2	LGI 1	*P* value	LGI 2	LGI 1	*P* value	LGI 2	LGI 1	*P* value
	Breakfast			Lunch			Snack			Dinner		
iAUC 0–1 h (mmol/L × min)	12.8 ± 4.6	29.1 ± 6.8	**0.02**	43.8 ± 8.0	55.1 ± 7.7	0.32	13.4 ± 3.1	22.4 ± 4.3	0.05	37.4 ± 7.8	61.7 ± 7.4	**0.03**
iAUC 0–2 h (mmol/L × min)	23.9 ± 8.1	54.8 ± 10.1	**0.008**	75.1 ± 14.0	81.1 ± 10.7	0.71	28.1 ± 6.8	37.1 ± 8.7	0.26	82.5 ± 17.0	128.7 ± 13.0	**0.02**
iAUC 0–3 h (mmol/L × min)	28.8 ± 9.4	62.5 ± 10.8	**0.009**	103.7 ± 21.0	104.6 ± 14.5	0.97				140.6 ± 24.9	174.2 ± 17.5	0.25
Incremental glucose peak value (mmol/L)	0.4 ± 0.1	1.0 ± 0.2	**0.002**	1.1 ± 0.2	1.3 ± 0.2	0.42	0.5 ± 0.1	0.6 ± 0.1	0.06	1.4 ± 0.2	1.9 ± 0.2	0.16
CV	4.88 (IQR: 4.05)	8.60 (IQR: 6.79)	**0.04**	5.40 (IQR:5.48)	6.21 (IQR:4.99)	0.35	3.96 (IQR: 2.28)	5.47 (IQR: 4.16)	0.05	7.51 (IQR:3.64)	8.54 (IQR:4.55)	0.15
24 h iAUC (mmol/L × min)	393.0 (IQR: 596.2)	511.9 (IQR:280.4)	0.51									

All values are mean ± SEM. iAUC was calculated using the trapezoid rule as area under the curve for glucose above baseline value.

*CV* coefficient of variation, *iAUC* incremental area under the curve, *IQR* interquartile range.

^1^All values of CV and 24 h iAUC are presented as median (IQR), and non-parametric *t* test was used.

*P* value < 0.05 are bolded in the table.

### Insulin response

There were no significant differences in the fasting concentrations of insulin prior to the consumption of the LGI 1 and LGI 2 biscuits at breakfast (*p*-value = 0.25). At breakfast, the incremental insulin response to LGI 2 biscuits were significantly lower than to the LGI 1 biscuits in volunteers (Fig. 3). The overall iAUC insulin response over 180 min was significantly lower for LGI 2 biscuits (*p*-value = 0.02).

## Discussion

The purpose of this study was to investigate the glycaemic effects of consuming two biscuits in the low GI range (i.e. 24 and 54) and its impact on postprandial glucose. Modifying the biscuits with functional ingredients was essentially to create a healthy, nutrient-dense, high-fibre low GI biscuit (LGI 2) that would favourably impact glucose metabolism and yet not increase overall energy intake. Therefore, this novel low GI biscuit (LGI 2) was created to be advantageous for body weight and glycaemic control.

The consumption of LGI 2 biscuits (containing 50 g available carbohydrates) resulted in a 56.4% reduction in glucose response and a concomitant 45% reduction in insulin response at breakfast. LGI 2 biscuits consumed as a mid-afternoon snack (containing 25 g available carbohydrates), showed a 24% reduction in glucose response, albeit not significant, but may be physiologically relevant. There was no effect on the second-meal glucose tolerance at standard lunch when LGI 2 biscuits were given. Repeating the analysis for the iAUC120 of lunch while controlling for the iAUC120 of breakfast did not change the conclusions reported in Table 3 (results not shown). This again confirms that there was no residual effect of the breakfast onto lunch. Similarly repeating the analysis for iAUC120 of dinner with the iAUC from 9 am to 6 pm did not change the conclusions as well (results not shown). Previous studies have reported a delayed postprandial response in blood glucose after a low GI breakfast/morning meal to the subsequent meal^6^, and at breakfast after a low GI dinner^16^. Our results revealed a new finding with the most prominent effect on second-meal glucose tolerance observed at the dinner meal. The metabolic basis of this finding remains uncertain, but it has been shown that insulin resistance is higher at night than in the morning or during the day^17,18^. This results in a diurnal variation in insulin resistance and plasma FFA concentrations. Since the second meal effect is related to suppression of plasma free fatty acid (FFA) concentrations^19^, it remains to be studied how consuming the LGI 2 biscuits affect the time course of plasma FFA concentration to the subsequent meal and over the course of the day.

The CV of the glycaemic response are measures used to describe the variability. It is measured by dividing the standard deviation of the raw glucose responses by their mean values for the period of observation. Percentage CV (% CV) during the LGI 1 breakfast was significantly higher than under the LGI 2 biscuit conditions (Table 3). Borderline differences in the percentage CV values was also observed during the snack consumption (Table 3). It is to be noted that these variability values are small. This is mainly attributed to the fact that both the LGI 1 and LGI 2 biscuits were low GI biscuits which are known to result in lesser glycaemic fluctuation than their high GI counterparts. Furthermore, all the subjects in this study were healthy individuals with no type 2 diabetes. Hence the difference in % CV observed was relative between the treatment biscuits used in this study. Higher variability of the LGI 1 biscuit suggests that it results in greater fluctuations of the blood plasma glucose which would stress the system increasing the risk of insulin insensitivity and diabetes risk in the long-term^20^.

The LGI 2 (GI 54) biscuits were almost negligible in fibre content. Some earlier studies have shown that certain soluble fibres consumed at a dose as low as 5.1 g in the first meal of the day exhibited postprandial effects immediately following the first meal, resulting in residual effects that blunt postprandial glycaemia after meals eaten several hours after fibre ingestion^21^. In our study, the addition of soluble fibre in LGI 2 biscuits made up one-fifth of the LGI 1 biscuit formulation. This proportion of fibre may contribute to the suppression of acute glucose elevation after ingestion at breakfast and at mid-afternoon, attributed to their ability to delay carbohydrate digestion and absorption from the gut by increasing the viscosity of the stomach and intestinal contents^22^. It is generally accepted that fats lower GR, and the type of fat used also affects carbohydrate metabolism^23^. However, one criticism of some low GI foods is the high fat content, which is particularly concerning for people with diabetes due to their risk of cardiovascular disease. Emerging evidence has shown that the addition of functional lipids during cooking of carbohydrate-rich staple foods may be an effective and practical strategy for improving glycaemic control^24^.

The differential patterns in glucose and insulin responses at breakfast may be possibly explained by the variation in the fat type used for our LGI 2 biscuits. In our study, we used an equal proportion of coconut oil to replace butter in LGI 2 formulation. Previous work by our team has shown that coconut oil incorporated in baked bread showed the greatest attenuation of GR compared to butter^24^. There was an attenuation in GR with the LGI 2 (coconut-oil based) biscuits compared to LGI 1 (butter-based). Coconut oil contains medium-chain triglycerides (MCTs) such as lauric acid and myristic acid, which could delay gastric emptying rates due their higher osmolarity^25^ and form amylose–lipid complexes resulting in resistant starch formation^26^. The use of simple dietary interventions, such as the addition of functional lipids during cooking of carbohydrate-rich foods may be an effective and practical strategy for improving glycaemic control. As biscuits were made with other functional ingredients, a combination of other factors could contribute to the reduction in glucose and insulin response. The partial replacement of sucrose/sugar with a low GI sweetener was to provide glucose-attenuation properties and yet not compromise on palatability and taste. Protein fortification involved the addition of a protein powder to increase the protein content of the LGI 2 biscuits. Bearing in mind that Asians consume a largely plant-based diet^27^, a plant-based protein was chosen for our modified LGI 2 biscuits. Besides increasing the total amount of dietary protein in the modified biscuits, the source of protein also determines their effectiveness in the regulation of postprandial glycaemia, by having superior glycaemic-reducing effects than that of animal protein^28,29^.

The knowledge generated from this study suggests that modifying a wheat-based product, such as biscuits, with functional ingredients (plant-based fat, soluble fibre, plant-based protein and low GI sweetener) may provide a viable option for innovative food products that can modify the post-meal glycaemic response while preserving pancreatic beta-cells, especially at breakfast, as observed in our findings. The novel low GI biscuit (LGI 2) has shown to favourably impact glucose metabolism, and further work needs to explore its impact on other metabolic biomarkers such as triglycerides.

The strengths of our study was the randomized crossover design where each subject serves as his control. The CGMS™ was an important tool used in this study for monitoring the glycaemic response of volunteers in the centre and at home. This is important, as it is likely to mimic a “real-world” situation than a laboratory-based study, which was also the aim of our present study. The uniqueness of the present study was to feed a standard diet that only differed in the type of biscuit consumed. This question is especially relevant today in light of the increasing burden of diabetes and the need for healthier food products that can be a nutritious addition to the everyday meal plan. Among the limitations of the study was that it was conducted in a small group of healthy young Chinese males so the generalisability of our findings to other populations e.g. prediabetics, abnormal blood glucose, needs to be examined in the future. Our sample population was modest, however, the within-subject crossover design reduced the between-subject variability in our study. Also, we did not measure metabolic biomarkers such as plasma lipids and biochemical indices of satiety and appetite, as this study was designed to be an exploratory study.

In conclusion, our study shows that even within the low GI range, the GI value matters in influencing postprandial glucose. The postprandial glycaemic response of two types of biscuits that fall within the low GI classification (GI 24 and 54) differed with the novel low GI biscuits (GI 24) showing a more suppressed postprandial glycaemic response. A simple strategy based on the approach of using alternative, functional ingredients may have an important role in dietary management for individuals at risk of T2D and cardiovascular disease.
